# Endophthalmitis after strabismus surgery: incidence and outcome in relation to age, operated eye muscle, surgical technique, scleral perforation and immune state

**DOI:** 10.1111/aos.14446

**Published:** 2020-06-13

**Authors:** Huibert J. Simonsz, Tina Rutar, Stephen Kraft, Alberta A.H.J. Thiadens, Manou R. Batstra, Robert M. Verdijk, Karin U. Loeffler, Guntram Kommerell, the Endophthalmitis‐after‐Strabismus‐Surgery Reporting Group, Mariette Swart‐van den Berg, Mary J Schooneveld, Lamberdina C.J.W Drunen, Luc Missotten, Gerold H Kolling, Marcel P.M. Tusscher, Yair Morad, Paolo Nucci, Scott E Olitsky, Lionel Kowal, Hessel G Eppinga, Frank Duivenboden, Nicoline E Schalij, José J. Malacara Hernandez

**Affiliations:** ^1^ Department of Ophthalmology Erasmus Medical Center Rotterdam the Netherlands; ^2^ Department of Ophthalmology Cataract and Laser Institute of Southern Oregon Medford OR USA; ^3^ Ophthalmology & Vision Sciences University of Toronto Toronto ON Canada; ^4^ Medical Immunology Reinier Haga Medical Diagnostic Center Delft the Netherlands; ^5^ Department of Pathology Erasmus Medical Center Rotterdam the Netherlands; ^6^ Department of Ophthalmology University Clinic Bonn Germany; ^7^ University Eye Clinic Freiburg Germany

**Keywords:** endophthalmitis, strabismus surgery, surgical contamination, antisepsis, complication, enucleation, scleral perforation, bacterial carrier state, immune deficiency

## Abstract

**Purpose:**

Identify risk factors for endophthalmitis after strabismus surgery (EASS) and relate these to incidence and outcome.

**Methods:**

Ophthalmologists, who had operated, diagnosed or treated EASS, completed a case record form with 71 questions in six domains: Preoperative, Surgery, Perforation, Postoperative, Outcome and Experts’ opinion. To estimate the age‐specific incidence per number of strabismus operations in the Netherlands during 1994‐2013, the age distribution of Dutch cases was compared with the age‐specific rates of strabismus surgery in the Dutch Registry of Strabismus Operations and with population data. Exploratory data analysis was performed. The immune state was evaluated in six patients. Five enucleated eyes were studied histopathologically.

**Results:**

None of the 26 patients (27 eyes with EASS) were between 9 and 65 years old, except for one patient with retinal haemorrhage followed by endophthalmitis. In the Netherlands during 1994‐2013, the rate of EASS was approximately one per 11 000 strabismus operations, but one per 4300 for children aged 0–3 and one per 1000 for patients 65 and older. Endophthalmitis was diagnosed on postoperative day 1–4 in children aged 0–3. In all 15 children aged 0–5, the 16 affected eyes were phthisical, eviscerated or enucleated. The involved eye muscle had been recessed in 25 of 27 cases. It was a medial rectus in 15 of 16 children aged 0–6. It was a lateral (6), inferior (2) or medial (1) rectus in elderly. Scleral perforation went unnoticed in all children (no record in three) and in two of seven elderly (no record in two). Histopathology showed transscleral scarring compatible with scleral perforation in four patients but, in a two‐year‐old girl who had EASS together with a transient medial rectus palsy, the sclera underneath the former suture tract was not perforated but did contain the long posterior ciliary artery.

**Conclusions:**

Endophthalmitis after strabismus surgery (EASS) affects children and elderly, with a grave outcome in young children. It occurs after recession of the medial rectus muscle in children, and it may occur without scleral perforation. Age and perforation are key determinants that interact with other factors that determine the occurrence and fulminance of EASS.

## Introduction

Medical action is and will always be associated with a certain risk. This also applies to strabismus surgery. One should not be misled by the fact that grave complications after strabismus surgery are, fortunately, very rare and one should not refer to strabismus operations as being almost risk‐free. As Knobloch & Lorenz (1962) and others have aptly noted, grave complications after strabismus surgery are more common than appears from the literature because, for obvious reasons, failures are rarely published. Knobloch & Lorenz sent a questionnaire to 1150 ophthalmic surgeons and got 324 responses, covering an estimated 300 000 strabismus operations. Of the 193 serious complications reported, endophthalmitis after strabismus surgery (EASS) accounted for 87, death from anaesthesia for 60, scleral necrosis for 32, retinal detachment for 8, retrobulbar haematoma for 4 and sympathic ophthalmia for 2. Two cases of bilateral EASS and two with sympathetic ophthalmia led to bilateral blindness. Unilateral blindness resulted from 68 cases of EASS, 8 cases of retinal detachment and 4 cases of retrobulbar haematoma.

Thirty years later, Simon et al. (1992) sent a questionnaire to 342 ophthalmic surgeons, 223 of whom responded. Scleral perforations with known retinal damage occurred in 728 of nearly 554 000 eye muscle procedures performed by 223 surgeons. Fourteen retinal detachments and only three cases of EASS were reported. Nine patients sustained visual loss. In a surveillance study twenty years later, Bradbury & Taylor (2013) received 60 reports of adverse events and complications in a period during which approximately 24 000 strabismus operations were performed in the United Kingdom. There was a single reported case of EASS in a child and one report of retinal detachment in an adult, suggesting that EASS is an exceedingly rare condition. However, in a similar surveillance study on endophthalmitis following cataract surgery by Kamalarajah et al. (2004), under‐reporting was estimated by independently contacting units with databases of vitreous cultures. The corrected incidence was almost twice as high as the incidence of endophthalmitis estimated with the collected reports. Under‐reporting of EASS may be more likely than after cataract surgery, because of the large discrepancy between the burden of strabismus and that of loss of an eye, especially in young children, and because of concern for litigation that may follow.

It is generally assumed that EASS only occurs after the sclera has been perforated by a needle.

First, how frequently do perforations occur? In prospective studies that employed postoperative funduscopy in consecutive strabismus operations, Morris et al. (1990) found one case of perforation (sclera and retina) in 67 patients (100 eyes), Noel et al. (1997) found 3 perforations and 14 funduscopic abnormalities without retinal perforation in 765 children (1129 recessions and 349 resections), Dang et al. (2004) found 6 perforations (sclera and retina) and 11 scleral penetrations without retinal perforation in 144 patients (217 eyes), Kaluzny et al. (1977) found 11 scleral penetrations with or without retinal perforation in 108 eyes, Taherian et al. (2004) found 10 perforations in 700 eyes (1121 muscles) and Surachatkumtonekul et al. (2009) found 15 perforations in 1095 patients (2195 muscles). In conclusion, perforations are two orders of magnitude more frequent than EASS. What other conditions must be met for EASS to develop? Is contamination of the needle or suture a rare event that could explain the rare occurrence of endophthalmitis after scleral perforation ?

On the contrary, Olitsky et al. (1998) and Carothers et al. (2003) found between 16% and 25% of needles or sutures to be contaminated after strabismus surgery. Rogers et al. (2011) found between 30% and 34% contaminated sutures or needles after strabismus surgery, with or without extra scrubbing of the eyelashes with 5% povidone–iodine. Saber Moghaddam et al. (2011) found a close similarity between the bacteriae cultured from the fornix before povidone–iodine antisepsis and those cultured from contaminated needles or sutures after strabismus surgery.

Conversely, is a scleral perforation a prerequisite for EASS to develop? In a series of six children (median age 2 years) with endophthalmitis and blindness after strabismus surgery, Recchia et al. (2000) found no anatomical changes of the sclera or the retina that were suggestive of a perforation, in histopathological examination of the enucleated eyes of two of the children.

In the current series of 26 patients (27 eyes) with EASS, the authors, most of whom were the operating ophthalmologists of the patients, conjoined their experiences and could thereby study its incidence and outcome in relation to the age of the patient, to the kind of eye muscle that was operated, to the surgical technique that was used and to whether scleral perforation had occurred.

## Methods

EASS occurred in a two‐year‐old girl at the Erasmus Medical Center Rotterdam in February 2005. Thereafter, a Case Record Form with 71 questions in 6 domains was developed (Table 1) and sent to ophthalmologists who had operated, diagnosed or treated a case of EASS, and to ophthalmic pathologists who had evaluated a specimen for histopathological diagnosis.

**Table 1:** Endophthalmitis After Strabismus Operation Questionnaire

Preoperative: History and diagnosis
1. What was the date of birth of your patient and what the date of surgery (if unknown supply age)?
2. What were the orthoptic diagnoses?
3. What was the best corrected preoperative visual acuity?
4. What was the spherical equivalent?
5. Did the patient have complaints, other than cosmetic?
6. The eye where endophthalmitis occurred was the right or left eye?
7. Peculiarities in the history of the patient? Had the patient recurring airway infections, recent airway infections, pre or dysmaturity, immunologic deficiency, allergy, asthma, diabetes mellitus?
8. Was the patient vaccinated for H. Influenzae group B?
9. Was the patient in the month preceding surgery healthy? If not specify.
10. Did the patient take any medication?
11. Did influenza, colds or airway infections occur in an epidemic fashion in the period that surgery was performed?
12. How often do you see minor postoperative infection in strabismus patients with concurrent airway infection?
13. In what month and weekday did surgery take place? Daycare or admission? Local or general anaesthesia?
14. Was it a reoperation of the affected eye?
15. How many operations did this eye have before, for strabismus, for cataract or else?
16. In case of strabismus surgery, which muscles had been previously operated on the affected eye?
17. What strabismus operation was performed now?
**Surgery: Disinfection, kind of operation, instruments and events**
18. Who performed the strabismus operation?
Could you indicate how many strabismus operations he or she performed yearly, for how many years?
19. Who assisted during this operation?
20. In case of a resident operating, who was involved in operating the affected muscle?
21. What was, most likely, used for antisepsis, povidone‐iodine or else? What concentration was used?
22. Was the disinfectant past expiration date or was it used long after opening? What did you disinfect?
In case the fornix was disinfected, how? Were both eyes disinfected before draping?
Was disinfection repeated for the second eye after the first eye had been operated?
23. What suture and needle was used?
24. Have you had problems with surgical instruments for strabismus surgery or their sterilization?
Has anything happened during this or other strabismus operations that may have influenced sterility?
**Perforation**
25. Have you ever noticed that scleral perforation occurred during strabismus surgery?
26. How did you notice scleral perforation in such a case?
27. Has a scleral perforation been noticed during the operation with the complication?
Has a scleral perforation or retinal bleeding been noticed by funduscopy? Did you notice loss of vitreous?
28. In case of perforation, what was its subsequent treatment: with antibiotics, by cryocoagulation?
29. If a scleral perforation was not noticed, how sure are you that none occurred?
30. If a scleral perforation was noticed, was funduscopy performed after or at the end of surgery?
31. Did you notice any peculiarities during surgery?
32. On what postoperative day does funduscopy routinely take place after strabismus surgery?
33. Did you ever notice a scleral perforation with funduscopy that had not been suspected during surgery? How often?
**Postoperative: Medication, signs, symptoms and microbiological culture**
34. What postoperative treatment was prescribed?
35. Was the eye patched in the 24 hr after surgery?
36. How was the eye patched?
37. Was the eye patched the entire day or just at night?
38. Did the parents indicate difficulty administering eye drops or do you suspect such difficulty?
39. Were you under the impression that the patient (adult) had taken his or her eye drops as prescribed?
40. Who first noticed symptoms that pointed towards endophthalmitis or retinal detachment?
How many days after surgery did these occur? What were these symptoms?
41. Date of first postoperative examination. What were the symptoms?
42. What were the signs? Was eye motility limited more than could be expected from the surgery itself?
43. Was the patient examined by an orthoptist, an ophthalmologist or a resident?
44. Date that the diagnosis of endophthalmitis was made. What were the symptoms?
45. What were the signs?
46. Was the patient examined by an orthoptist, an ophthalmologist or a resident?
47. Was is possible to take a vitreous tap for culture at the time? Has a vitreous tap indeed been taken for culture?
48. Was Gram staining performed and what was the result?
49. What bacteria were cultured? Were Gram and culture results communicated to the ophthalmologist immediately?
50. Were antibiotics administered intravitreally? Were steroids administered intravitreally?
51. Were antibiotics administered by a different route?
52. Has other medication been administered?
**Outcome: Treatment, complications and visual outcome**
53. Did a cyclitic membrane develop? How many days postoperatively did miosis start?
54. What surgery or other measure was then carried out?
55. Did complications result from these operations or from other measures?
56. When was the last examination?
57. What was the visual acuity at that point?
58. Had phthisis occurred?
**Expert’s opinion: Risks, prevention, patient information, perforation, residents**
59. Is too little attention given to these complications during ophthalmology residency?
Is too little attention given to these complications during orthoptic training?
60. In the Netherlands, strabismus patients are often first examined postoperatively by an orthoptist only.
Do you now think all first postoperative exams should include an ophthalmological examination?
63. Have you become more reluctant in letting residents operate?
64. Can the choice who operates, ophthalmologist or resident, be influenced by the patient or by the child’s parents?
65. Have you become more reluctant to operate on the better eye in case of amblyopia?
66. Do you agree to: ‘A perforation cannot occur if you can see the point of the needle at all times?’
67. Do you agree to: ‘An endophthalmitis can only occur after a perforation?’
68. How should, in your opinion, information about the operation be given to patients and parents?
Who should, in your opinion, give this information?
69. Indicate items that should be specifically mentioned to the patients or their parents.
70. Did you extend the information given to patients and their parents after the complication occurred?

### Case record form

First, we made an inventory of all potential risk factors for EASS. Presumed risk factors were categorized in five domains according to the phase of the disease, applying to situations and circumstances before, during and after surgery. At a meeting of paediatric ophthalmologists, vitreoretinal surgeons and orthoptists devoted to EASS in Rotterdam in May 2006, several cases were discussed in detail and the evidence for and against many of the presumed risk factors was discussed. Questions were subsequently formulated for the Case Record Form within each domain: Preoperative, Surgery, Scleral perforation, Postoperative, Treatment and Outcome, Experts’ opinion (Table 1).

### Inclusion

From 2005 onwards, we contacted paediatric ophthalmologists and asked whether they had seen similar patients. First, the strabismus surgeons at all university clinics in the Netherlands and Belgium were contacted. As most operations for strabismus are performed by general ophthalmologists in the Netherlands, we also contacted ophthalmologists in most regional hospitals where strabismus surgery is performed, directly or indirectly. A similar procedure was attempted in Belgium, Germany and the UK. Calls to contribute cases were done at international meetings and in the newsgroup of the American Association for Pediatric Ophthalmology and Strabismus.

Patients were included when their strabismus had been operated or their EASS had been diagnosed, treated or evaluated by the participating ophthalmologist, orthoptist or ophthalmic pathologist. Patients in whom retinal haemorrhage had preceded EASS were included but analysed separately. The Case Record Form was filled out by the participating ophthalmologist, orthoptist or ophthalmic pathologist.

### Age‐specific rate of EASS per number of strabismus operations (exploratory data analysis)

We found comparatively high rates of EASS in children and in elderly patients, with no cases occurring between the ages of 9 and 65, apart from a 14‐year‐old girl who had a retinal haemorrhage after a myopexy of the medial rectus as the primary event. Strabismus surgery is carried out more frequently in children. Therefore, the age‐specific incidence of EASS had to be related to the age‐specific incidence rate of strabismus operations. To estimate these, on the one hand a well‐defined population was needed with most of the cases of EASS identified over a long period of time. On the other hand, a good estimate of the age‐specific incidence rate of strabismus operations in that population was needed over that same period of time. Seven cases of EASS had been identified in the Netherlands over the 20‐year period 1994–2013. In reality, more cases, adults in particular, may have occurred during that period. It seems unlikely, however, that more children were affected in that period, because information about such patients would have popped up in the numerous discussions we had in the Netherlands in many meetings devoted to the subject and at congresses with presentations on the subject, between 2005 and 2013.

To calculate the age‐specific incidence rate of strabismus operations in the Netherlands over 1994–2013, data were combined from the Dutch Registry for Strabismus Operations (consulted 25 March 2015), from incidence studies of strabismus operations in the United Kingdom and Canada, and from demographic population data.

First, the age distribution was determined of 7679 strabismus operations registered in the Dutch Registry for Strabismus Operations over an 8‐year period from 2007 to 2014. This registry, aimed at monitoring quality of care, is open for ophthalmologists in the Netherlands, to compare their results of strabismus surgery with the average results of strabismus surgery in the Netherlands. It was started in 2007 and is not mandatory yet, but increasing numbers of ophthalmologists register their strabismus operations in the registry. The 7679 age‐specific, registered strabismus operations were compared with the age composition of the population in the Netherlands from 2007 to 2014 (consulted 25 March 2015) to find the age‐specific incidence rate of registered strabismus operations.

To estimate what fraction of the strabismus operations in the Netherlands had been registered in the Dutch Registry for Strabismus Operations in the 8‐year period from 2007 and 2014, we compared the annual incidence of strabismus operations per age group found by Arora et al. (2005) for Scotland (age 0–14: 8.8), for England and Wales (age 0–16: 7.8) and for Ontario (age 0–14: 7.2; age 0–16: 6.6) in 2000, and those found by Heng et al. (2013) in 2010 for Scotland (age 0–15: 7.5), for Wales (age 0–14: 5.7) and for England (age 0–14: 6.4), with the annual incidence of strabismus operations registered in the Dutch Registry for Strabismus Operations for similar age groups: For age 0–14 this was given as: 1.83, for age 0–15: 1.75, and for age 0–16: 1.69. Accordingly, the annual incidences of strabismus operations in the Netherlands that are registered in the Dutch Registry for Strabismus Operations were approximately a fourth of those found in the United Kingdom and Canada.

The incidence rate of strabismus operations in the Netherlands is not likely to be very much different from those in Scotland, England, Wales and Ontario and, therefore, we assumed that approximately one fourth of the strabismus operations had been registered during the 8‐year period from 2007 to 2014. Accordingly, we multiplied the incidence rates of age‐specific, strabismus operations registered in the Dutch Registry for Strabismus Operations by four to estimate the overall age‐specific incidence rates of strabismus operations in the Netherlands. Finally, these rates were multiplied by the age composition of the population in the Netherlands in the 20 years from 1994 to 2013 (CBS Statistics Netherlands, 2015) to estimate the age‐specific number of strabismus operations in the Netherlands over the 20‐year period from 1994 to 2013, during which seven cases of EASS had occurred in the Netherlands, three in small children and four in elderly.

### Bacterial species, histopathology and immune state

The results of Gram stains, vitreous cultures and conjunctival cultures were analysed. Patients 5, 6, 8, 10, 19 and 22 were invited to the Sophia Children’s Hospital at the Erasmus Medical Center Rotterdam for assessment of their immune state. After a physical examination, antibodies against *S. pneumoniae* capsular polysaccharides and other immunological parameters were assessed in blood samples.

Enucleated eyes of cases 5, 6, 8, 11 and 18 were studied histopathologically. In patients, 5 and 8 additional sections were made of the original histopathological specimens, to identify or exclude scleral perforations.

### Institutional review board

As the study was retrospective and included patients treated in the past anonymously, assessment of the study was not considered necessary by the Institutional Review Board of the Erasmus Medical Center in Rotterdam. However, for publication of the histopathological specimens of enucleated eyes of cases 5, 6, 8 and 11 who were minor at the time of the enucleation, written permission was obtained from them and, when they were still minor, from their parents.

## Results

Twenty‐three ophthalmologists and one orthoptist from the United States, Mexico, Canada, Australia, Italy, Israel, Germany, Belgium and the Netherlands reported on 26 patients (27 eyes). The data compiled from the Case Record Forms of the patients are summarized in Table 2.

**Table 2:** Summarized data of the 26 patients (27 cases) with EASS

No	Age	Year surg	Comorbidity & previous surgery affected eye	Diagnosis	Acuity affected eye	Acuity unaffected eye	Se aff eye	Se unaff	Involved rectus	Operation	Perfor noted	Primary diagnosis	Diagn at	Anterior chamber	Culture	Subsequent findings	Vitrectomy	Subsequent surgery	Last known state	Period postop
1	0.9	2011	siblings with respiratory illness at time of surgery	esotropia	fixated and followed	fixated and followed	+6	+6	medial	recession	no	endophthalmitis	day 2	hypopyon	pneumococ		day 2: cult & AB, day 6: vitrect with lensect & silic oil		phthisis	1 month
2a	1	2004	none known	esotropia					R medial	recession	no	endophthalmitis RE	day 2	hypopyon	pseudom aer	day 2: fever, eyelid edema	day 2: cult & AB, day 3: vitrect, day 14: vitrect, phaco & silic oil	day 121: vitrect for cyclitic membrane	LP	14 years
2b	1	2004	none known	esotropia					L medial	recession	no	endophthalmitis LE	day 2	hypopyon	pseudom aer	day 2: fever, eyelid edema	day 2: cult & AB, day 3: vitrect, day 14: vitrect, phaco & silic oil		NLP	14 years
3	1.5	2011	brother with influenza at time of surgery	esotropia	fixated and followed	fixated and followed	+1.5	+1.5	medial	recession	no	endophthalmitis	day 3	hypopyon	H influenzae		day 5: vitrectectomy		phthisis	3 months
4	1.7	2009	twice: bilat med r recessions; bilat lat r resections	residual eso	central steady maintained fix	central steady maintained fix	+2	+2	lateral	re‐resection	no	endophthalmitis	day 4	plasmoid	pneumococ	day 4: cyclitic membrane	day 4: vitrectomy with lensectomy		LP, small eye	2 years
5	1.7	1994	prematurity, asthma from age 7 months, excema as baby	esotropia	fixated and followed	fixated and followed			medial	recession	no	endophthalmitis	day 2	fibrin	negative	day 7: sinusitis, day 10: cornea ulcer, lens opacity	day 55: vitrectomy	day 119: enucleation	enucleation	4 months
6	2.4	2005	recurrent resp inf; lacr duct obstr; otitis media & NVI palsy 3 months preop; cold week preop	esotropia	central steady unmaintained fix	central steady maintained fix	+1	+1	medial	recession	no	endophthalmitis	day 2	hypopyon	H influenzae	day 21: cyclitic membrane	day 28: vitrectomy with lensectomy & silicone oil	1.5 years: enucleation	enucleation	14 years
7	2.7	2007	prematurity	esotropia	0.8	1.0	+1.5	+1.25	medial	recession		endophthalmitis	day 3	hypopyon	coag neg staph	day 8: cyclitic membrane	day 9: vitrectomy with lensectomy	day 15: enucleation	enucleation	day 15
8	3.1	2004	none known	esotropia	0.75	0.75	+5	+5	medial	recession	no	endophthalmitis	day 1	hypopyon	negative	day 15: US: vitr opacities & thickened choroid		day 284: enucleation	enucleation	9 months
9	3.5	1970		esotropia	good	good			medial	recession		endophthalmitis	day 3			cyclitic membrane			phthisis	6 months
10	4.1	1999	prematurity, psychomotoric retardation	esotropia	1.0	1.0	+1.25	+1.75	medial	recession	no	endophthalmitis	day 2	hypopyon	coag neg staph	day 27: cyclitic membrane	day 7: ant chamber flushed, day 17: vitrect with silic oil	1 year: silic oil out & EDTA scraping	phthisis	6 years
11	4.5	1959		esotropia	central steady unmaintained fix	central steady maintained fix	+1.5	+1.5	medial	recession	no	endophthalmitis	day 21	uveitis	no culture	day 13: cyclitic membrane		1.1 years: enucleation	enucleation	1.1 year
12	5.5	1955		esotropia	good	good			medial	recession		endophthalmitis	day 2						phthisis	few months
13	5.5	1960	circumcision together with strabismus operation	esotropia	good	good			medial	recession	no	endophthalmitis	day 7						phthisis	years
14	6.6	1979	prematurity, med r recession & lat r resection	residual eso	0.3	0.9			medial	recession	no	endophthalmitis	day 14	clear	haemolyt strept				VA 0.3	6 weeks
15	6.5	2005		esotropia	1.0	1.0	0	0	medial	recession	no	endophthalmitis	day 3	hypopyon	staph aur		day 3: vitrectomy		VA 1.0	2 weeks
16	9.5	1991	pharyngitis 1 week preop	exotropia	1.0	1.0	0	0	lateral	recession	no	endophthalmitis	day 5	hypopyon	pneumococ	day 10: cyclitic membrane	day 10: vitrectomy	day 13: evisceration	evisceration	7 weeks
17	14.6	1997	cataract surgery at age 3	secondary eso	0.5	1.0	+15	0	medial	myopexy	no	sutures visible with retinal hemorrhage	day 1	clear	no culture		day 3: sutures removed: retinal detachment & end developed	day 28: vitrect, encircling & silic oil	phthisis	9 years
18	65.3	2005	strabismus surgery 9 years preop	exotropia	1.2	1.2			lateral	recession		endophthalmitis	day 2					1 year: enucleation	enucleation	1 year
19	65.8	2007	strabismus surgery 1 year preop	residual exo	1.0	1.0	+1	+3.25	lateral	recession	yes	endophthalmitis	day 4	cells	negative		2.7 years: vitrectomy for macular pucker	4.3 years: cataract surgery	VA 0.8	4.4 years
20	66.5	1995	hypertension, well‐controlled asthma	exotropia	1.0	1.0			lateral	recession		endophthalmitis	day 8	cells	staph aur	retinal‐vitreous opacity, possibly abcess	day 12: scleral defect closed surgically; retinal scar	11 years: cataract surgery	VA 1.0	11 years
21	66.9	1996		consec exo	1.0	0.1			lateral	recession	yes	perfor: cryocoag; day 1: vitr hemorrh	day 1	vitreous hemorrhage	no culture	day 1: laser for perfor; day 10: vitrect for hemorrhage	day 183: cat surg		VA 0.25	several years
22	70.9	2008	diabetes mellitus II, coronary bypass 16 years preop	exophoria	1.0	1.0	+2.75	+2.5	lateral	recession	yes	endophthalmitis	day 4	cells	negative				VA 0.9	2 months
23	72.5	1983	cataract surgery 1 year preop	hypertropia	1.0	1.0			inferior	recession	no	endophthalmitis	day 8	hypopyon	pseudom aer	day 8: cyclitic membrane	day 9: vitrectomy with intraocular lens removal		LP	day 20
24	74.7	1995	Graves, narrow‐angle glaucoma, laser iridotomy & trabeculoplasty	hypotropia	1.0	1.0	‐1	0	inferior	recession	yes	endophthalmitis	day 7		coag– staph epid		day 8: vitrectomy		VA 0.32	11 months
25	74.9	2015	caroticocav fistula, NVI palsy, Hummelsheim & med r recession 1 year preop	consec exo	1.0	0.9	+1	‐0.50	lateral	hangback recession	no	endophthalmitis	day 6	cells & flare	staph epid	macular pucker, atrophy in macula and periphery			VA 2/300	18 months
26	85.5	2003	cataract surgery 2 months preop	NVI palsy	1.0	1.0			medial	recession	yes	endophthalmitis	day 14	low‐grade uveitis	no culture	day 14: exsudative ret detachment			VA 0.8	months

The patients are listed according to age. A blank space signifies unknown or unavailable data. ‘age’ denotes age at surgery, ‘SE aff eye’ spherical equivalent affected eye, ‘SE unaff’ spherical equivalentunaffected eye, ‘involved rectus’ rectus muscle which operation caused endophthalmitis, ‘perfor’ perforation, ‘diagn at’ postoperative day the diagnosis was made, ‘cult’ culture of the vitreous in the acutestage, ‘period postop’ period between strabismus operation and last examination, ‘bilat’ bilateral, ‘r’ rectus, ‘med’ medial, ‘lat’ lateral, ‘inf’ inferior, ‘resp inf’ respiratory infection, ‘pre/postop’ pre‐/postoperatively, ‘caroticocav’ caroticocavernous, ‘eso’ esotropia or ‐phoria, ‘exo’ exotropia or ‐phoria, ‘consec’ consecutive, ‘m’ muscle, ‘R/LE’ right/left eye, ‘day 1’ first postoperative day, ‘AB’ antibiotics,‘hypop’ hypopyon, ‘vitr’ vitreous, ‘vitrect’ vitrectomy, ‘lensect’ lensectomy, ‘sil oil’ silicone oil, ‘end’ endophthalmitis, ‘phaco’ phacoemulsification, ‘cyclit memb’ cyclitic membrane, ‘IOL’ intraocular lens,‘N/LP’ no/light perception, ‘VA’ decimal visual acuity, ‘pneumococ’ S. pneumoniae, ‘pseudom aer’ P. aeruginosa, ‘H influenzae’ H. influenzae, ‘staph aur’ S. aureus, ‘staph epid’ S. epidermidis, ‘haemolytstrept’, haemolytic streptococcus, ‘coag neg staph’ and ‘coag– staph’ coagulase negative Staphylococ.

All but one of the treating and operating ophthalmologists and one treating orthoptist are co‐authors. In none of the 26 cases, legal action had been taken, but in three cases complaints have been submitted.

Data about cases of EASS were collected from 2005 to 2018. The information was gathered directly by the surgeon from the patient’s record in all cases except in cases 9 and 12 where the patient’s paper record was no longer available. The ophthalmologist remembered the essential findings in these patients in detail, however. Case 18 was reported by the ophthalmologist who examined the enucleated eye histopathologically. Case 11 was reported by an ophthalmologist after review of the ophthalmic pathological records and has been published previously (Huang et al. 2011).

Patients 17 and 21, who were 14 and 66 years old, had a retinal haemorrhage after a perforation as primary event and developed vitreous haemorrhage, retinal detachment and endophthalmitis later. In case 17, myopexy of the medial rectus muscle caused a retinal haemorrhage. After subsequent removal of the suture a vitreous haemorrhage occurred and retinal detachment and endophthalmitis developed. In case 21, cryotherapy was performed after scleral perforation, but a vitreous haemorrhage developed the following day which was treated by vitrectomy on the third postoperative day.

### Age‐specific rate of EASS per number of strabismus operations (exploratory data analysis)

**Fig. 1:**
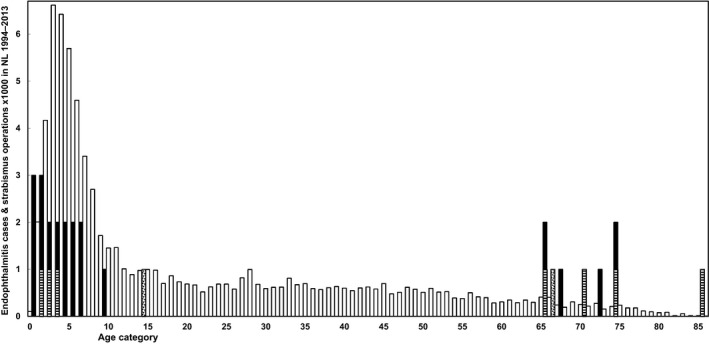
Age distribution of the 26 patients (27 eyes). Black bars represent cases of EASS that occurred abroad or in the Netherlands before 1994. Striated bars represent cases that occurred in the Netherlands over the 20‐year period from 1994 to 2013. This is set against a background of thousands of strabismus operations per year of age (white bars) in the Netherlands in the 20 years from 1994 to 2013, derived and extrapolated from the Dutch Registry for Strabismus Surgery, population data and strabismus‐surgery surveillance studies. Two cases with retinal perforation with haemorrhage primarily and retinal detachment and EASS secondarily are represented by dotted bars.

The ages of the remaining 24 (25 eyes) patients with EASS as primary event were either 9 years or younger, or 65 years or older (Fig. 1). The age‐specific incidence rates of strabismus operations in the Netherlands in the 20‐year period from 1994 to 2013 were estimated as described in the Methods section. Both the age‐specific number of strabismus operations in the Netherlands and the seven cases that occurred in the 20‐year period from 1994 to 2013 are shown in Fig. 1, together with the other 19 patients (20 eyes) that occurred in the Netherlands before 1994 or occurred outside the Netherlands.

The overall rate of EASS in the 20‐year period 1994–2013 in the Netherlands was estimated at 1:10 968 strabismus operations. It was higher for young and for old age groups, however. On the basis of the observed three young children with EASS in the Netherlands during 1994–2013, it was estimated at 1:3141 for age 0–2 and 1:6439 for age 0–4. On the basis of the observed four elderly with EASS in the Netherlands during 1994‐2013, it was estimated at 1:959 for 65 years and older (Table 3).

**Table 3:** Age‐specific rate of EASS per number of strabismus operations

Age group	Odds 1:	Age group	Odds: 1:
0–1	2114	65–98	959
0–2	3141	66–98	1141
0–3	4299	67–98	1006
0–4	6439	68–98	924
0–5	8337	69–98	859
0–6	9869	70–98	755
0–7	11004	71–98	1005
0–8	11904	72–98	895
0–9	12477	73–98	755
0–10	12963	74–98	676
0–11	13451	75–98	1139
0–12	13788	76–98	898
0–13	14086	77–98	714
0–14	14413	78–98	532
0–15	14745	79–98	414
0–16	15072	80–98	315

On the basis of the number of cases that occurred in the Netherlands over the 20‐year period from 1994 to 2013 and estimated age‐specific number of strabismus operations in the Netherlands over the same period, both depicted in Fig. 1, the age‐specific rate of EASS per number of strabismus operations can be calculated. The overall rate of EASS in the 20‐year period 1994–2013 in the Netherlands was 1:10,968 strabismus operations. In the table the observed age‐specific rate of EASS per number of strabismus operations are listed for children within subsequent age groups from birth (left) and for elderly patients within subsequent age groups until death (right).

### Clinical data from case record forms

The data collected from the Case Record Forms are summarized below for each domain: Preoperative, Surgery, Scleral perforation, Postoperative, Treatment and Outcome, Experts’ opinion. Items that are relevant for presumed risk factors for EASS are worked out in some detail.

Pivotal items have been listed in Table 2 for quick comparison.

### Domain preoperative

Three children had been born prematurely. Four of the 8 children aged 0–2 years, or their siblings, had had an upper airway infection or influenza shortly before or during surgery. Only one patient, 22, had diabetes mellitus type II. Patients 14, 17 and 21 had amblyopia with more than 2 logMAR lines of difference in visual acuity. All 15 children under age 9 were operated for esotropia. Refractive error was not different from what would have been expected to occur in the 16 children and the 9 elderly patients. Patient 16 had had unilateral cataract surgery at age 3 and that eye was hyperopic and amblyopic. The better eye was operated only in patient 21.

### Domain surgery

Fourteen of the 17 strabismus operations in children and 3 of the 9 strabismus operations in the elderly patients were bilateral. Patient 13 had had a circumcision with strabismus surgery in the same session of surgery. Case 2 had bilateral EASS. The involved eye muscle – the operation of which caused the endophthalmitis – was recessed in 25 out of 27 cases. A myopexy and a resection of the involved muscle were done in the other two.

In 15 of 17 children, the involved eye muscle was a medial rectus. A lateral rectus re‐resection caused endophthalmitis in patient 4 aged 1 who had had previous medial rectus recessions and previous lateral rectus resections. A lateral rectus recession caused endophthalmitis in patient 16 aged 9.

Among 9 elderly patients, surgery on the lateral rectus caused endophthalmitis in 6 cases, on the inferior rectus in 2 cases and on the medial rectus in 1 case. A hang‐back recession was done in case 25. Among the elderly patients, the affected eye had been operated on previously in 6 out of 9 cases. Most operations were performed under general anaesthesia, patients 21 and 26 were operated in local anaesthesia; in case 18, this was unknown. Six out of 21 patients were operated on a Friday.

For preoperative antisepsis, povidone–iodine was used in 15 out of 16 cases and cetrimide in one case. In 5 cases 10% povidone–iodine solution was used, in three cases 1%, in four cases 5% and in the remaining cases this was unknown. The conjunctival fornices were rinsed in 6 cases. In cases of bilateral surgery, disinfection was repeated before operating the second eye in 2 out of 15 cases. Few surgeons had paid attention to the expiration date of the povidone–iodine solution, whether it had been diluted or not to 1%, for instance, or to the date of opening of the bottle.

Sutures had been Vicryl in 17 of 17 cases and this was unknown in 9 cases. Among the 10 specified needles were S‐4, S‐29 and TG‐100. No abnormalities regarding sterility were noticed during surgery. In case 13, strabismus surgery was performed together with a circumcision.

### Domain postoperative

All but 2 patients received antibiotic eye drops and ointment immediately after surgery, in 4 cases this was unknown. The parents of cases 2, 4, 6 and 10 had difficulty administering the antibiotic eye drops or ointment postoperatively, because of lack of cooperation of the child.

The diagnosis of EASS was made in children under 4 on postoperative days 1–4 and in older patients up to 21 days after surgery. The anterior chamber contained a hypopyon in 10 out of 18 eyes of the 17 children. It contained fibrin, plasmoid, ‘uveitis’ in 3, it was clear in 2, and in 3 cases this was unknown. In adults, hypopyon was seen in only 1 of 7 cases, in 2 cases this was unknown. Bilateral EASS developed in case 2 aged 1. He had fever and bilateral eyelid oedema together with the endophthalmitis.

According to the parents of most young children, their child initially played during the first postoperative day, to become less active or photophobic in the course of the second postoperative day. At the first postoperative visit, the orthoptist or ophthalmologist noticed a red and/or troubled eye in most children. Two adults presented with a painful eye. Patients 6 and 14 had a transient palsy of the involved eye muscle.

*Streptococcus pneumoniae* and *Haemophilus influenzae* were the most frequent pathogens in children, whereas *S. aureus, S. epidermidis* and *P. aeruginosa* occurred both in children and in elderly patients. One culture result had been noted as *haemolytic streptococcus* without further details. The bilateral EASS in case 2 aged 1 was caused by *P. aeruginosa*.

### Domain perforation

Scleral perforation had not been noticed by the surgeons during surgery in 14 of 14 children (no record in 3), but it had been noticed in 5 of 7 elderly patients (no record in 2).

The histopathology of the enucleated eyes of cases 5, 8, 11 and 18 showed transscleral scarring, compatible with prior full‐thickness scleral perforation (Figs 3, 4, 5). In the histopathology examination of case 6, the layers of the sclera beneath the former tract of the suture, that was removed 2 days after EASS was diagnosed, 5 days postoperatively, were found to be undisturbed, but they did contain a scleral channel with nerves and a blood vessel, presumably the long posterior ciliary artery (Fig. 6).

**Fig. 2:**
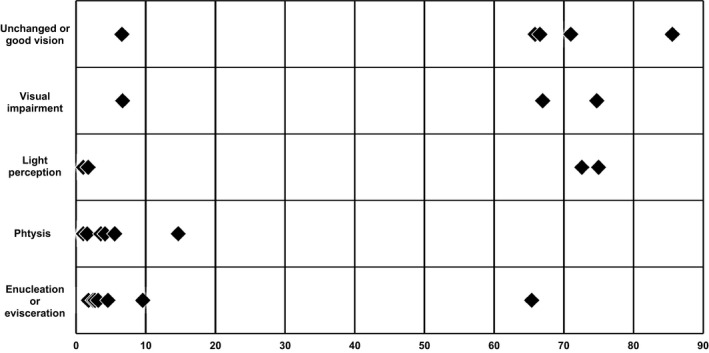
Histopathology of the enucleated eyes of case 8. Transscleral scarring with cicatrization tissue protruding into the eye, compatible with prior full‐thickness scleral perforation. The location of the presumed perforation was 5.5 mm from the limbus of the eye, and the eye measured 17 9 16 mm. Haematoxylin‐eosin stain, original magnification 25x.

**Fig. 3:**
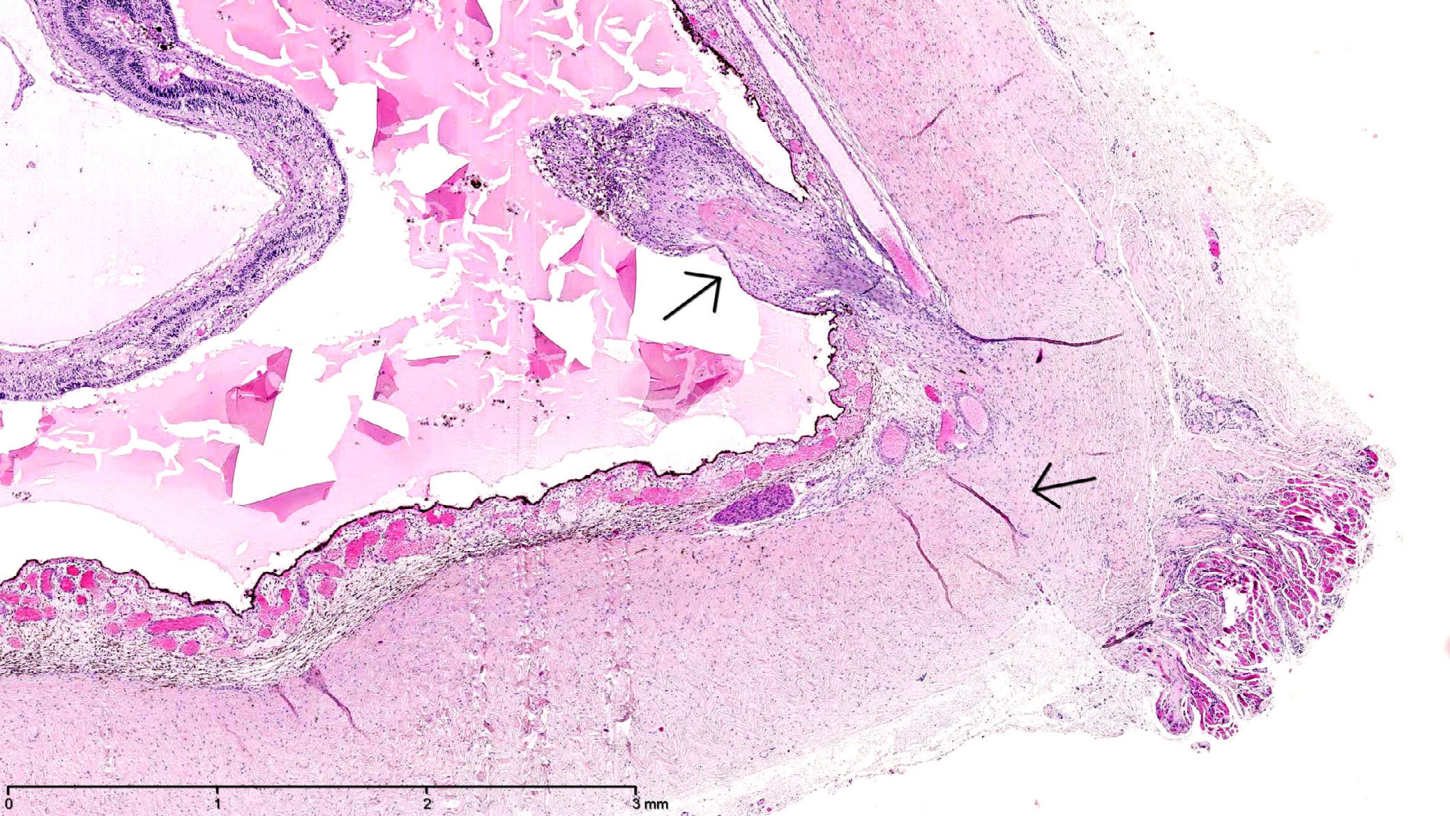
Histopathology of the enucleated eyes of case 8. Transscleral scarring with cicatrization tissue protruding into the eye, compatible with prior full‐thickness scleral perforation. The location of the presumed perforation was 5.5 mm from the limbus of the eye, and the eye measured 17 9 16 mm. Haematoxylin‐eosin stain, original magnification 25x.

**Fig. 4:**
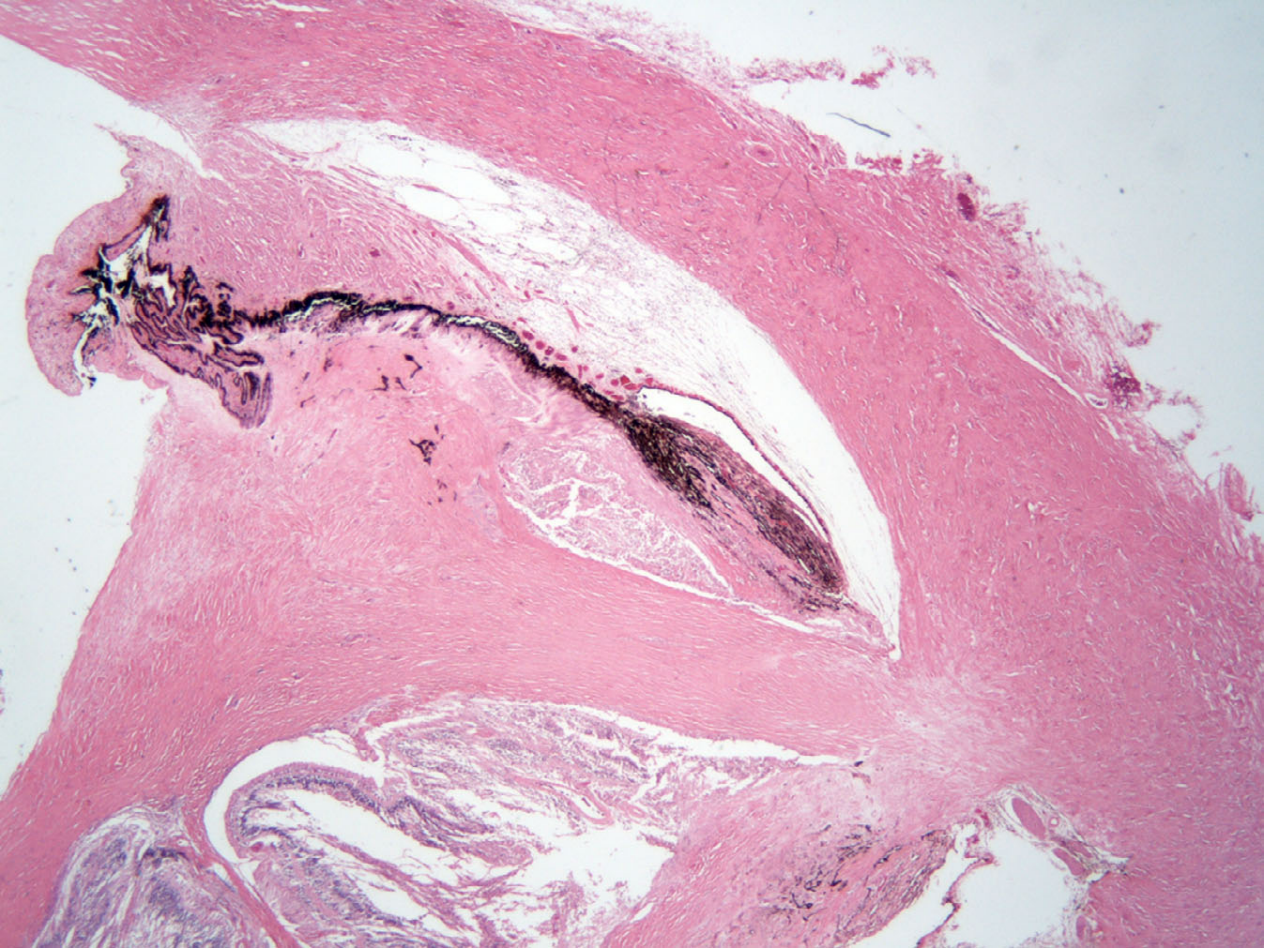
Histopathology of the enucleated eyes of case 8. Transscleral scarring with cicatrization tissue protruding into the eye, compatible with priorfull‐thickness scleral perforation. The location of the presumed perforation was 5.5 mm from the limbus of the eye, and the eye measured 17 x 16 mm.Haematoxylin‐eosin stain, original magnification 25x.

**Fig. 5:**
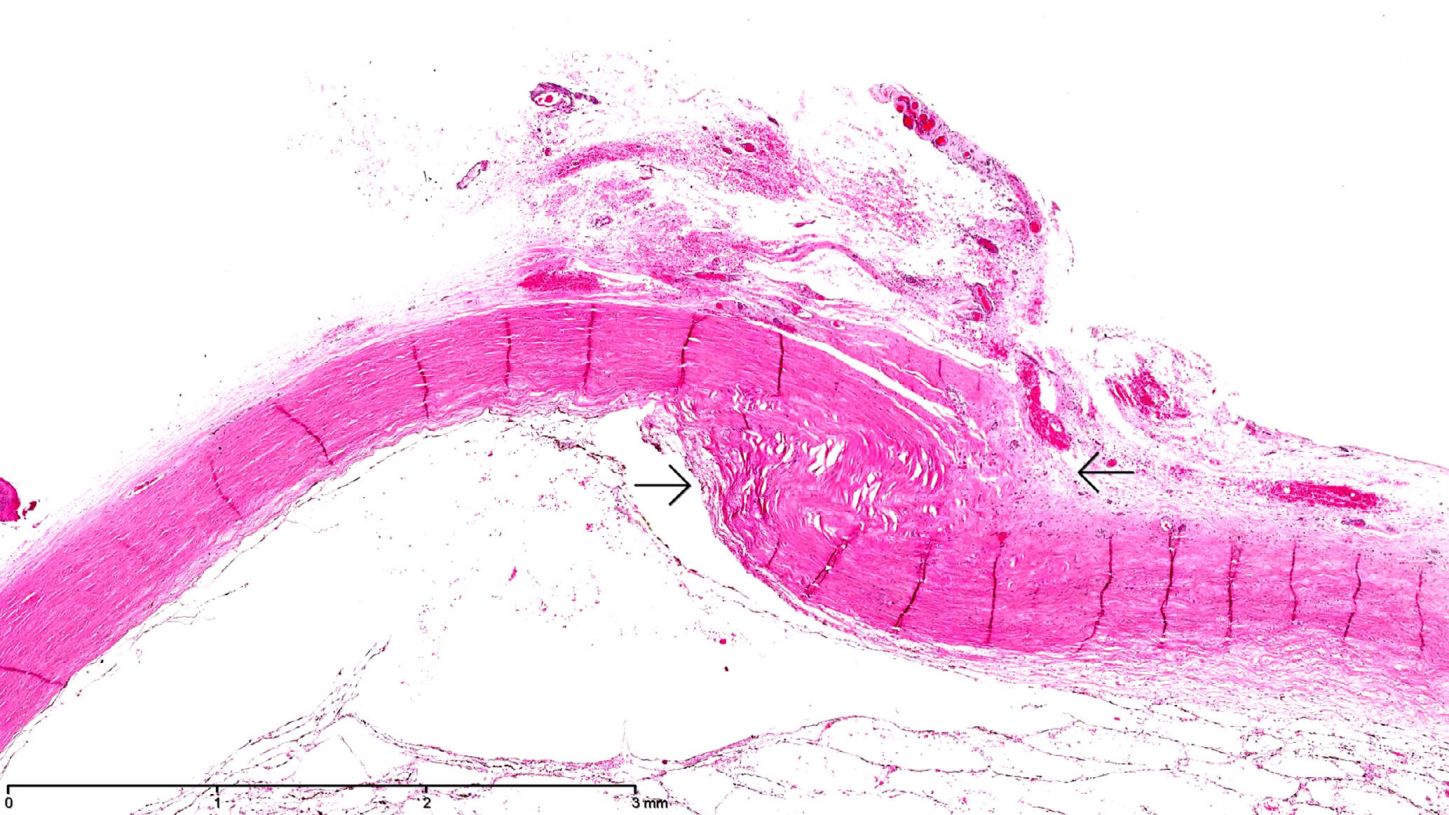
Histopathology of the enucleated eye of case 18. Transscleral scarring with cicatrization of the sclera, compatible with prior scleral perforation. Haematoxylin‐eosin stain, original magnification 12.5x.

In the histopathology examination of case 6, the layers of the sclera beneath the former tract of the suture, that was removed 2 days after EASS was diagnosed, 5 days postoperatively, were found to be undisturbed, but they did contain a scleral channel with nerves and a blood vessel, presumably the long posterior ciliary artery (Fig. 5). This two‐year‐old girl had developed a right otitis media 3 months before the strabismus surgery with fever up to 41.9°C. Two weeks later esotropia developed, she grasped past objects and stumbled. The paediatric neurologist suspected that a right sixth nerve paresis could have arisen from the otitis media and petrositis. A CT‐scan showed aerated petrosal cells, however. She had had a lacrimal duct obstruction of the right eye in the first year of life that had resolved spontaneously. She had been treated for recurrent upper airway infections with Ventolin in the months after the otitis media. A recession of the right medial rectus muscle and a resection of the right lateral rectus muscle was performed for a now concomitant esotropia, together with a posterior tenotomy of the inferior oblique muscle of both eyes. On examination on the third postoperative day, the child was photophobic and had a red, painful eye that looked troubled. No adduction of the right eye was possible for several days, indicative of complete medial rectus palsy, adduction to return to normal in the weeks after. The anterior chamber contained cells, flare and fibrin. An encapsulated, mucoid *H. influenzae* type A was cultured from the vitreous. Weekly ultrasound examinations showed a contracting vitreous, but no retinal detachment or another indication of a perforation. Three weeks postoperatively a cyclitic membrane developed, causing pain at night. A lensectomy with vitrectomy with silicone oil were performed on the 27th postoperative day. After removal of the lens and cyclitic membrane, the retina was found to be attached without retinal defects, but it appeared necrotic nasally. The painful and hypotonic eye was ultimately enucleated 17 months after the strabismus operation. A chronic purulent discharge of the socket developed. Culture of the discharge and deep throat cultures at age 5 again showed an encapsulated, mucoid *H. influenza,* the same species that had caused the EASS, but multi locus sequence typing of the two strains showed they were not identical. In a subsequent immunological assessment of a panel of 6 antibodies against *S. pneumoniae* capsular polysaccharides, those against serotypes 1, 3, 4, 9 and 23 were undetectable, whereas that against serotype 5 was 0.19 µg/ml (Table 4). Most children have undetectable antibody levels against one or two serotypes, but against 5 out of 6 serotypes is unusual at age 5. As we surmised that an immune defect could contribute to the development of endophthalmitis, patients 5, 8, 10, 19 and 22 were also invited for immunological assessment. Undetectable levels of antibodies were found against only 1 of 6 serotypes in 3 patients, and against 2 of 6 serotypes in patient 9 (Table 4). In case 6, after immunization with *H. influenzae* (Act‐Hib^®^) and *S. pneumoniae* capsular polysaccharides (Pneumovax^®^), the purulent discharge subsided. In subsequent repeat immunological assessment, the levels of the 6 antibodies against *S. pneumoniae* capsular polysaccharides were adequate (Table 4).

**Table 4:** The immune state was evaluated in patients 5, 6, 8, 10, 19 and 22

Patient	Age	Serotype 1	Serotype 3	Serotype 4	Serotype 5	Serotype 9	Serotype 23
5	15	0.32	3.35	3.83	0.10	1.92	0.22
6 pre imm.	5	<0.10	<0.10	<0.10	0.19	<0.10	<0.10
6 post imm.	5	3.62	2.96	1.44	0.39	0.29	0.83
8	6	0.19	2.67	1.44	0.39	0.29	0.83
10	12	0.18	< 0.10	0.30	0.37	< 0.10	0.52
19	65	0.19	0.43	0.11	0.54	> 7.77	0.90
22	71	0.69	0.58	0.10	0.74	2.73	1.37

Levels of IgG antibodies (µg/ml) against polysaccharide capsule of *S. pneumoniae* serotypes 1, 3, 4, 5, 9 and 23 were measured, years after the endophthalmitis had occurred. Levels above 0.35 µg/ml are generally considered protective. Patient 6 had undetectable levels against all but one serotypes. She was immunized with H. influenzae (Act‐Hib^®^) and *S. pneumoniae* capsular polysaccharides (Pneumovax^®^). For patient 6, levels before and after immunization are shown.

### Domain outcome

In 7 children, a cyclitic membrane developed, whereas in 3 more cases vitrectomy was performed within a week, precluding the development of a cyclitic membrane. A cyclitic membrane developed in only one of the elderly patients. Vitrectomy was performed in most patients. In all 15 children under age 6 (16 eyes), the affected eye was enucleated, eviscerated or became phthisical. A visual acuity better than 0.25 was attained in 2 out of 4 children aged 6–14 and in 7 out of 9 elderly patients.

**Fig. 6:**
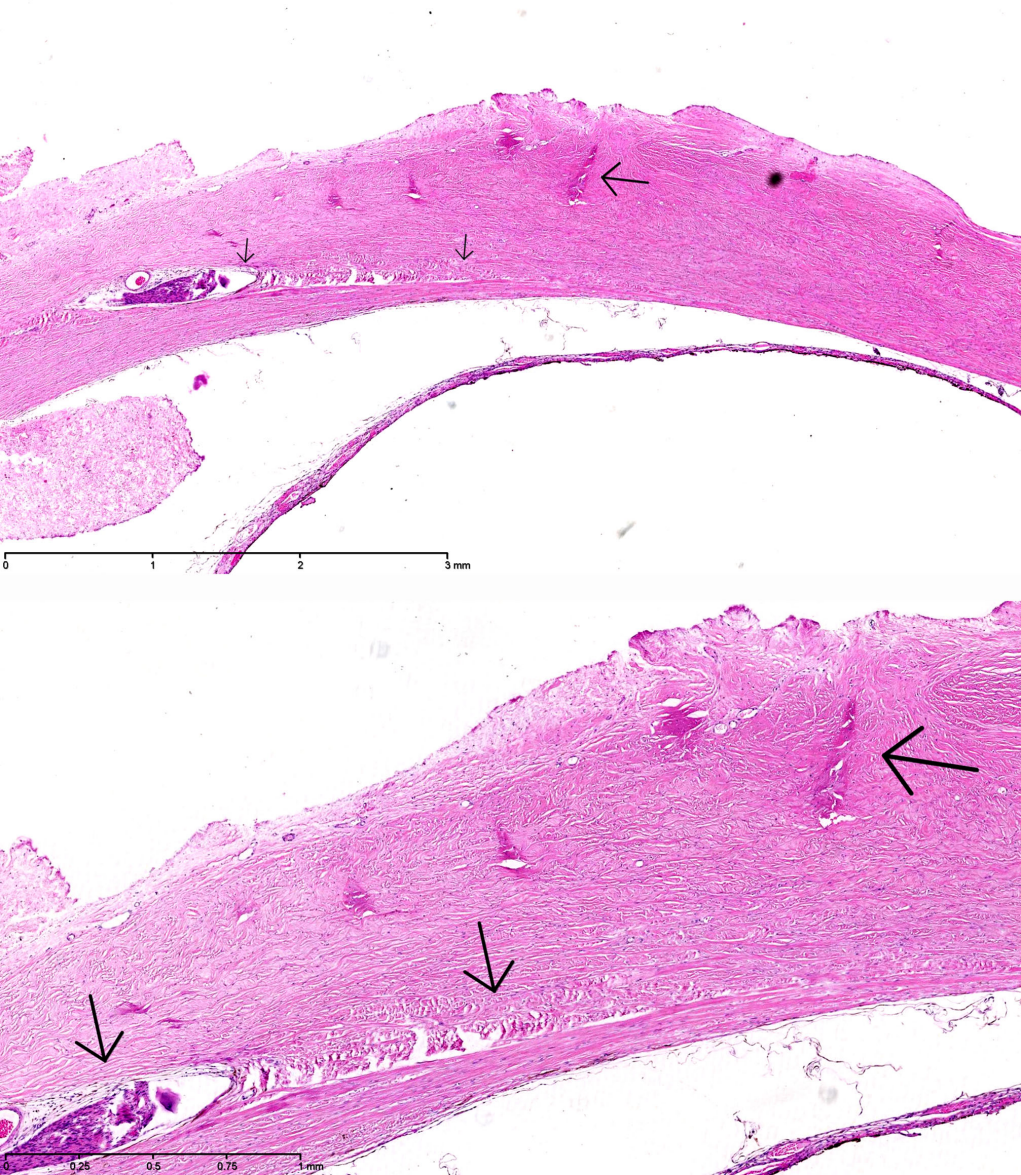
Histopathology of the enucleated eye of case 6. The layers of the sclera beneath the former tract of the suture (upper panel, long arrow), that was removed 2 days after EASS was diagnosed, were undisturbed (upper panel, short arrow)), but underneath the former suture tract (lower panel, long arrow) was a scleral channel with nerves and a blood vessel (lower panel, short arrow), presumably the long posterior ciliary artery, which may have carried the infection forward into the eye. Haematoxylin‐eosin stain, original magnification 25x (upper panel), 50x (lower panel).

### Domain experts’opinion

At the end, the Case Record Form contained statements regarding EASS to obtain experts’ opinion from those who had operated or treated a case of EASS themselves. The main results are summarized in Table 5. More than half of the ophthalmologists had become more reluctant to operate on the better eye in case of amblyopia, but they had not become more reluctant to let residents operate. Almost all used an information sheet, in addition to oral information, that included the risk of losing vision or the eye. Most did not believe that EASS occurs only after a perforation, nor that a perforation cannot occur if the tip of the needle remains visible through the sclera. Most thought that the first postoperative exam should include an examination by an ophthalmologist. Half thought that too little attention was given to this complication during residency or orthoptic training.

**Table 5:** To obtain experts’ opinion, the ophthalmologists and the one orthoptist who had operated or treated a case of EASS were asked to agree or not with statements regarding EASS

Have you become more reluctant to operate on the better eye in case of amblyopia?	10 of 17
Have you become more reluctant in letting residents operate?	2 of 12
Can the choice who operates, ophthalmologist or resident, be influenced by the child’s parents?	9 of 13
For informed consent use information sheet in addition to oral information	14 of 16
Who should, in your opinion, give this information?	ophthalmologists: 8
	orthoptists: 1
	both: 7
Oral information or information sheet should mention risk of losing vision or eye	12 of 16
Did you extend the information given to patients and their parents after the complication occurred?	8 of 14
Do you agree with: ‘Endophthalmitis only occurs after perforation’	1 of 16
Do you agree with: ‘Perforation cannot occur if you see point of needle’	5 of 16
First postoperative exam should include examination by ophthalmologist	12 of 16
Too little attention given to these complications during ophthalmology residency or orthoptic training	9 of 17

The two figures following each statement represent the number in agreement with the statement and the total number of respondents.

## Discussion

By bringing many cases of EASS together we could relate its incidence and outcome to age, operated eye muscle, surgical technique, scleral perforation and immune state.

None of the patients were between 9 and 65 years old, except for one patient with a retinal haemorrhage followed by EASS. In almost all children the EASS resulted from a medial rectus recession. Half of the young children or their siblings had had an upper airway infection. In children, scleral perforation had never been noticed during surgery. The EASS was diagnosed on postoperative day 1‐4 in children under 5 years of age. A 2‐year‐old girl developed EASS and a transient medial rectus palsy 3 days after a medial rectus recession. Histopathology after enucleation one and a half year later showed that the sclera underneath the former suture tract had no signs of full sclera perforation, but it did contain the long posterior ciliary artery. Its channel through the sclera may have allowed bacteriae to enter into the eye. In all children under age 6 the eye was enucleated, eviscerated or phthisical. A visual acuity better than 0.25 was attained in 2 out of 4 children aged 6–14 and in 7 out of 9 elderly patients.

Endophthalmitis after strabismus surgery (EASS) between age 6 and 65 years is rare. Bialasiewicz et al. (1990) reported on a 10‐year‐old boy who had a purulent endophthalmitis 3 days after a medial rectus myopexy, like our case 17 had at the age of 14. In myopexy surgery, the muscle belly is anchored to the sclera with nonresorbable sutures. The sutures must be placed deep in the sclera, otherwise the sutures are subsequently pulled out of the sclera. Bilateral EASS is also rare. Knoblauch & Lorenz (1962) had 2 bilateral cases among 72 in their survey.

The predilection for EASS to develop in young children and in elderly could be related to the absence of IgG mediated immunity in early childhood and its decline in senescence. The rapid and relentless course of EASS in young children would be compatible with this supposition. An additional explanation could be that adolescents and adults voice complaints and seek medical attention earlier and may therefore be diagnosed in earlier stages of the disease.

Scleral perforation had not been noticed by the surgeon in any of the children (no record in 3), but it had in 2 of 7 elderly patients (no record in 2). Scleral perforation is noticed when the resistance suddenly drops when the needle is passed through the sclera, or when blood or vitreous appear on the sclera. It is possible that the drop of resistance is less outspoken in children due to their more elastic sclera. It is also possible that, in children, a noticed and treated perforation does not evolve into an endophthalmitis and, hence, such cases were not included in this study.

In case 6 the sclera underneath the former tract of the suture, which was removed 5 days postoperatively, had not been perforated, but it did contain the long posterior ciliary artery, as found histopathologically after enucleation. This finding confirms the conclusion by Recchia et al. (2000) that ‘the development of EASS neither requires nor implies that full perforation of the sclera has occurred’. In case 6, a complete, transient medial rectus palsy occurred together with the EASS, compatible with bacterial contamination of the suture and subsequent myositis. The scleral channel of the long posterior ciliary artery may have carried bacteriae from the contaminated suture into the eye.

Case 6 was a carrier of a mucoid, encapsulated *H. influenzae* type a, found both at age 2 in the eye and at age 5 in the socket, nose and throat. At age 5, she had undetectable levels of antibodies against capsular polysaccharides of all but one *S. pneumoniae* serotype. Some of the capsular polysaccharides of *S. pneumoniae* are homologous with those of *H. influenzae* (Lagergård & Branefors 1983)). Mucoid bacteria produce slime that forms a biofilm on the conjunctiva. They thereby survive antibodies and antibiotics. Half of the children carry *H. influenzae* at age 5, but these are mostly noncapsular strains of *H. influenzae* (Katosova 1994). Before the introduction of Hib vaccination in the Netherlands in 1993, about 700 children annually had a severe *H. influenzae* b infection. After vaccination started this number decreased to 17 in 2001, but it surged again, inexplicably, in children aged 0‐4 in 2005 (Rijks Instituut voor de Volksgezondheid, accessed 6 January 2017; McVernon et al. 2004), the year that our case 6 had an invasive H. influenzae type a infection and EASS.

In 14 of the 15 children aged 0–6 (16 eyes), the EASS was caused by a medial rectus recession. In case 4 it was caused by a re‐resection of a lateral rectus muscle. In a recession, the sclera where the sutures are placed is thinner than the sclera at the original insertion and the long posterior ciliary artery lies more superficially in that area of the sclera. Surachatkumtonekul et al. (2009) found that all 15 perforations among 2195 operated eye muscles in his study occurred during a recession. In our study, evidence is not very strong, however, as in the Dutch Registry for Strabismus Operations (16) almost 82% of the operations in children were recessions and between 80% and 85% of the operated eye muscles were medial rectus muscles.

The predilection for EASS to occur after medial rectus surgery in children (15 of 16 children aged 0–6) may be related to reflux from the lacrimal sac. The medial rectus muscle lies underneath and between the lacrimal puncta. The nasolacrimal duct can be obstructed functionally by an upper airway infection. Four of the 8 children aged 0–2, or their siblings, had an upper airway infection at the time of surgery. In the study by Recchia et al. (2000), 3 out of 6 children had had an upper airway infection. Good et al. (1990) described three children with endophthalmitis after cataract surgery with either nasolacrimal duct obstruction or upper airway infection at the time of surgery. Kam et al. (2014) found nasolacrimal duct obstruction more often among patients who developed endophthalmitis after cataract surgery as compared to those who did not develop endophthalmitis. Speaker et al. (1991) found the same bacteria in the nose as in the vitreous aspirate in the majority of 17 patients with endophthalmitis after cataract surgery.

For preoperative antisepsis, 10% povidone–iodine solution was used in five cases, 5% in four cases and 1% in three cases. To determine whether differences in effectiveness existed between concentrations, we repeated the study that Ferguson et al. (2003) had performed in adult patients operated for cataract, in children operated for strabismus. In a multicentre, randomized controlled trial (30), we rinsed the conjunctiva either with 5% or with 1.25% povidone–iodine prior to strabismus surgery, in children aged 1–5. After induction of anaesthesia, cultures were taken from the nose, from the conjunctiva and, at the end of surgery, from the reattached eye muscle with cut‐off sutures. As has been found by Olitsky et al. (1998), Carothers et al. (2003) and Rogers et al. (2011), many cultures from reattached muscles with cut‐off sutures were positive. In several cases, the same bacteriae were found on the reattached eye muscle as had been found in the nose before the operation began. In some of these cases, however, cultures taken from the conjunctiva before the operation began were either negative or carried other bacteriae, indicating that the surgical field had been recontaminated during surgery by reflux from the lacrimal sac. Emptying the lacrimal sac by compressing it with a cotton‐tipped applicator before rinsing the conjunctiva with povidone–iodine effectively reduced the number of positive cultures from the reattached muscle in that study (Li et al. 2014).

### Study limitations

The small sample size limited our ability to perform statistical analysis in this case series. The associations between age and incidence per number of strabismus operations and between age and outcome could, hence, not be statistically secured, nor could the comparatively high EASS rates in young children and in elderly patients be linked to the absence of IgG mediated immunity in early childhood and its decline in senescence, also because the immune status of most patients was unknown. Although in children, EASS occurred almost exclusively after medial rectus recession, this association could not be statistically secured as in the Netherlands around that time more than 80% of the operations in children were recessions and more than 80% concerned medial rectus muscles. Future longitudinal studies with larger sample sizes are required to confirm that age, surgical technique, operated muscle, perforation and immune state are associated with incidence and outcome. Awaiting these results, we can only offer our own policies for consideration: We now postpone strabismus surgery in children under age 6, not only in case of fever, but also if eyes are tearing or nose is running. During preoperative antisepsis, we empty the lacrimal sac by compressing it with a cotton‐tipped applicator before rinsing with povidone–iodine. During a recession, we identify the long ciliary posterior artery and steer clear of it with the needle. In many European countries a child is first examined postoperatively by an orthoptist: an orthoptist can check with a retinoscope whether the red‐reflex is bright and symmetrical in case of unusual complaints.

**Fig. 7:**
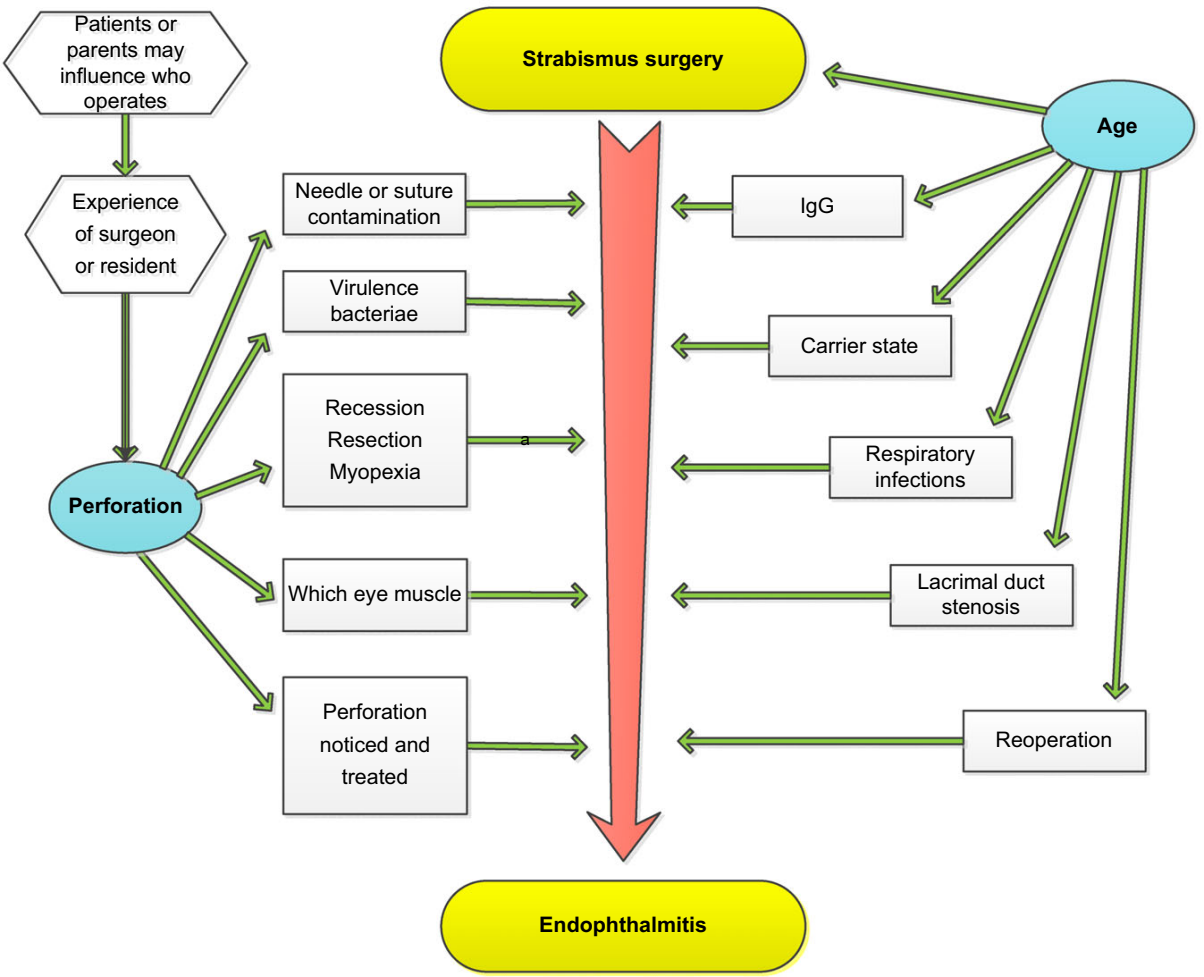
Perforation and age influence most parameters that determine the occurrence and fulminance of EASS. As a model for further research, the causal relations of these two in relation to their target conditions is depicted

Age and perforation are key determinants that interact with other factors that determine the occurrence and outcome of EASS. As a model for further research, the causal relations of these with their secondary determinants are depicted, provisionally and open to discussion, in Fig. 7.

## Acknowledgements

We are greatly indebted to Nicolaas G. Hartwig for examination and immunologic assessment of 6 cases (Department of Infectious diseases, Erasmus Medical Center, Rotterdam, the Netherlands); Cornelia M. Mooy for histopathological examination of 3 cases (Department of Pathology, Erasmus Medical Center, Rotterdam, the Netherlands) and her suggestion that instead of a perforation, the scleral channel of the long ciliary artery serves as entry port for endophthalmitis; John Sloper for suggesting that reflux from the lacrimal puncta could cause endophthalmitis after medial rectus surgery in particular; Marinus J.C. Eijkemans for statistical analysis (Department of Public Health, Erasmus Medical Center, Rotterdam, the Netherlands); Willem B. van Leeuwen for microbiological assessment of case 6 (Department of Medical Microbiology, Erasmus Medical Center, Rotterdam, the Netherlands). We are also greatly indebted to the patients and their parents, for their continued support of this study.

## Appendix

Mariette Swart‐van den Berg (Ophthalmology, Leiden University Medical Center, Leiden, the Netherlands); Mary J. van Schooneveld (Ophthalmology, University Medical Center Utrecht, Utrecht, the Netherlands); Lamberdina C.J.W van Drunen (Ophthalmology, Hospital Rivierenland, Tiel, the Netherlands); Luc Missotten^†^ (Ophthalmology, University Clinic, Leuven, Belgium); Gerold H. Kolling (phthalmology, University Clinic, Heidelberg, Germany); Marcel P.M. ten Tusscher (University Hospital, Brussels, Belgium); Yair Morad (Pediatric Ophthalmology Service, Assaf Harofeh Medical Center, Tel Aviv University, Tel Aviv, Israel); Paolo Nucci (Eye Clinic, San Giuseppe Hospital Milan, University of Milan, Milan, Italy); Scott E. Olitsky (Ophthalmology, Children's Mercy Hospitals and Clinics, Kansas City, MO, USA); Lionel Kowal (phthalmology, Centre for Eye Research Australia, Melbourne, VIC, Australia); Hessel G. Eppinga (Ophthalmology, Deventer Hospital, Deventer, the Netherlands); Frank van Duivenboden (Ophthalmology, Hospital Group Twente, Almelo, the Netherlands); Nicoline E. Schalij (Ophthalmology, Leiden University Medical Center, Leiden, the Netherlands); José J. Malacara Hernandez (Clínica Oftalmológica, León de los Aldama, Mexico).
